# Roman Catholic Inebriates and Their Religion

**Published:** 1903-10-10

**Authors:** 


### Roman Catholic Inebriates and their Religion.

An inmate of the Victoria Inebriate Homes at Brentry, near Bristol, recently escaped from the place, clad only in a nightshirt, and as this belonged to the institution, he was taken up on a charge of theft. He had, it was said, feigned illness, and had been allowed to stay in bed, and when alone had picked the locks of the day-room and the front door and escaped. His defence was that he was a Roman Catholic, and had asked in vain several times for the ministrations of a Catholic priest. Apparently the bench did not accept this as a sufficient explanation for his breaking bounds, and sentenced him to a month's imprisonment. Yet it is evident from the last report of the Homes that there is some trouble over the religious question. At the end of 1902 the number of Catholic inmates of the Brentry Homes was 50, of whom, however, a considerable number were due to leave during the first half of the current year. On this point the warden, Mr. H. N. Burden, says in his report: "Compared with that (*i.e.* the table showing the religious belief of the inmates) for the preceding year a very marked decrease in the number of Roman Catholic inmates will be noticed. The cause of this marked decrease is that a large number of the cases admitted from outside sources to fill empty beds in 1901 turned out to be Roman Catholics." Further on he adds:—"In my opinion, it would be well if the board were to make arrangements for the care of Roman Catholic cases in some other institution. The London County Council will not permit Roman Catholics to become inmates of the Reformatory established by that body, but has arranged for them to be sent to other institutions, and I am of opinion that it would be a distinct advantage if the board could see its way to do so also."

Without attempting to penetrate all that lies behind these statements, one cannot but see that the Warden thinks that the presence of Roman Catholics in the Homes is undesirable. Whoever is to blame for this state of things, the result is regrettable. Inebriates will be found among Roman Catholics as well as among Protestants, and unless the church to which they belong has the means and the will to build a special refuge for them, they must be sent to a general inebriate reformatory when, by consent or compulsion, they are withdrawn from ordinary life in order to cure them of their failing. It is natural that the governor of such a reformatory should feel a keener interest in the weaknesses, physical and moral, of the inmates than in their different religious beliefs. His influence should be sufficiently strong to prevent more dispeace among people of different creeds than there is in the outside world, where, after all, Roman Catholics, Churchmen, and Dissenters jog along comfortably enough. But these creeds should be strictly respected. If the prisoners in our gaols are privileged to have the services of the clergy of the church to which they profess adherence it seems hard that the inmates of an inebriate reformatory should be less fortunate. The drunkard on the road to reform particularly needs the consolations and encouragement of religion. The emotional repentance of a half-reclaimed drunkard may not be a very powerful force for good, but if turned back on itself for lack of sympathy, may be a very strong reactionary influence. It is quite comprehensible that the lack of a priest's ministrations really did so affect the escaping inmate of the Brentry Homes—who was likely enough an excitable and ill-balanced man—that it made his confinement there unendurable. Whether this be so or not, it is to be hoped that the publicity given to the matter will tend to bring about some arrangement between the authorities of the Roman Catholic Church and those of the Homes which will prevent such an excuse being again given by a refractory or truant inmate.

